# Pneumothorax After Shoulder Arthroscopy: A Rare Complication of Rotator Cuff Repair Surgery

**DOI:** 10.7759/cureus.36774

**Published:** 2023-03-27

**Authors:** Konstantinos Kateros, Emmanouil Skotidis, George D Bablekos, Maria Vlachou, Konstantinos Giatroudakis, Olga Theodorolea, Spyridon P Galanakos

**Affiliations:** 1 First Orthopaedic Department, G. Gennimatas General Hospital, Athens, GRC; 2 Biomedical Sciences, Occupational Therapy & Nursing Department, University of West Attica, Athens, GRC; 3 Orthopaedic Department, Primary Health Care Corporation, Athens, GRC

**Keywords:** anesthesia, risk factors for pneumothorax, pneumothorax (ptx), rotator cuff repair surgery, arthroscopic shoulder surgery

## Abstract

Shoulder arthroscopy is considered a very safe surgical procedure; however, there are possible complications that are prevalent or devastating. This article presents a 52-year-old woman scheduled for elective arthroscopic rotator cuff repair under general anesthesia in the lateral decubitus position. Postoperatively, the patient experienced dyspnea, chest pain, and oxygen desaturation, and a diagnosis of pneumothorax was made. A chest tube was inserted promptly and the patient was discharged in a good condition, experiencing an uneventful follow-up for the next four months. Knowledge of this uncommon complication may enable both surgeons and anesthesiologists to set up preventive and early therapeutic management.

## Introduction

Shoulder arthroscopy is a frequently performed and accepted technique for a wide variety of shoulder pathologies [1]. Despite the increasing use of this safe procedure over the past 20 years, common and rare complications still exist [2]. These complications are classified as preoperative (related to surgeon experience, patient selection or positioning, to anesthesia), intraoperative (neurovascular injury, hardware failure, fluid extravasation, fracture), or postoperative (infection, thromboembolic events, hemarthrosis, persistent pain, stiffness, heterotopic ossification) [3]. Moreover, uncommon, respiratory complications such as pneumothorax have been reported in patients undergoing the aforementioned procedure [4]. Although this complication during shoulder arthroscopy is rare [5], its presence might be life-threatening and prompt diagnosis and treatment are mandatory.

## Case presentation

A 52-year-old female, reporting a history of pain in her right shoulder associated with limited function and unresponsive to one-year conservative medical treatment, was admitted to our department. The patient’s body mass index was 22.6 kg/m^2^ (specifically, 167 cm in height and 63 kg in weight). The patient reported a medical past of hypertension and hyperlipidemia, both being under medication. Medical history of pulmonary diseases (such as bronchial asthma, chronic obstructive pulmonary disease, tuberculosis) or allergies, as well as smoking and/or alcohol habits, was not reported. In addition, the patient had no prior invasive procedures on the shoulder. Physical examination and diagnostic imaging were both consistent with a symptomatic full-thickness rotator cuff tear of the supraspinatus tendon. The patient was classified as status I, according to the Anesthesiologist's Physical Status classification system. The preoperative laboratory findings were within normal ranges, and a chest radiograph showed no abnormality.

In the operating room, the patient was connected to a pulse oximeter, a capnograph, an electrocardiograph, and an automated blood pressure cuff for continuous monitoring of her vital signs. The patient underwent general anesthesia, with intubation being accomplished under direct vision and she was placed in a lateral decubitus position (LDP).

The anesthesia was managed with volume-controlled ventilation (VCV), with an intraoperative tidal volume of 7-8 mL/kg of the ideal body weight, while the administered tidal volume (VT) was specifically at 500-525 mL. The respiratory rate (RR) fluctuated between 12 and 14 breaths/min and the end-tidal carbon dioxide (EtCO_2_) was maintained between 28 and 36 mmHg. Positive end-expiratory pressure (PEEP) values fluctuated between 4 and 5 cm H_2_O and the FiO_2_ value was at 0.5 (50%) in oxygen. The maximum airway pressure values, during arthroscopy with the patient in LDP, were between 20 and 27 cm H_2_O, the P plateau was between 15 and 20 cm H_2_O while the lowest saturation in the same position was at 95%. General anesthesia was induced by using Propofol, Fentanyl, and Rocuronium. Sugammadex was administered to reverse neuromuscular block. For anesthesia maintenance desflurane was used in a mixture of oxygen/nitrous oxide 50%.

To perform the arthroscopic procedure, three 5 mm stab standard portal incisions, such as anterior, posterior, and lateral, were used. Moreover, an anterolateral portal presents an alternative working and/or diagnostic role. The procedure consisted of subacromial decompression, acromioplasty, bursectomy, and double-row rotator cuff repair using suture anchors (Arthrex, Naples, FL, USA). A 4.5 mm shaver blade, a 5 mm burr as well as an electrocautery suction device were all the instruments used to accomplish acromioplasty. Particularly, the shaver blade and burr were both consecutively positioned through an anterolateral portal incision. In addition, the acromioclavicular joint excision was performed through the anterior portal. There were no difficulties with trocar placement and bleeding was minimal. During the procedure, the pump pressure was maintained at 50 mmHg and it was kept stable throughout the operation. The total surgical time was 100 minutes. During the procedure, the patient's vital signs were stable (oxygen saturation was 99-100%, systolic blood pressure < 90 mmHg, heart rate from 65 to 70 beats/min) and airway pressure remained unchanged.

**Figure 1:**
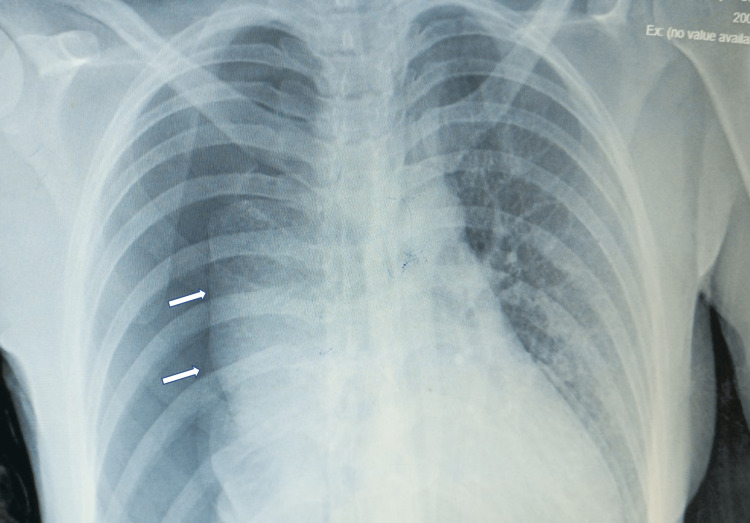
The chest X-ray shows the right pneumothorax. The two white arrows note the pneumothorax border.

Upon completion of the operation, extubation was achieved with the patient being completely in a status of self-respiration and consciousness. The patient was transferred to the post-anesthesia care unit, where her vital signs were as follows: blood pressure at 120/60 mmHg, heart rate at 70 bpm, RR at 14 breaths/min, body temperature at 36.2^o^C, and oxygen saturation at 98% with the use of an oxygen mask. Fifteen minutes after recovery, the patient developed dyspnea and chest pain. The oxygen saturation was decreased to 92%, despite the use of a mask for oxygen delivery at a flow rate of five liters per minute, accompanied by a decrease in blood pressure such as 73/47 mmHg along with a simultaneous increase in heart rate at 110 bpm. An urgent portable chest radiograph was performed showing a right-sided pneumothorax (Figure 1).

**Figure 2:**
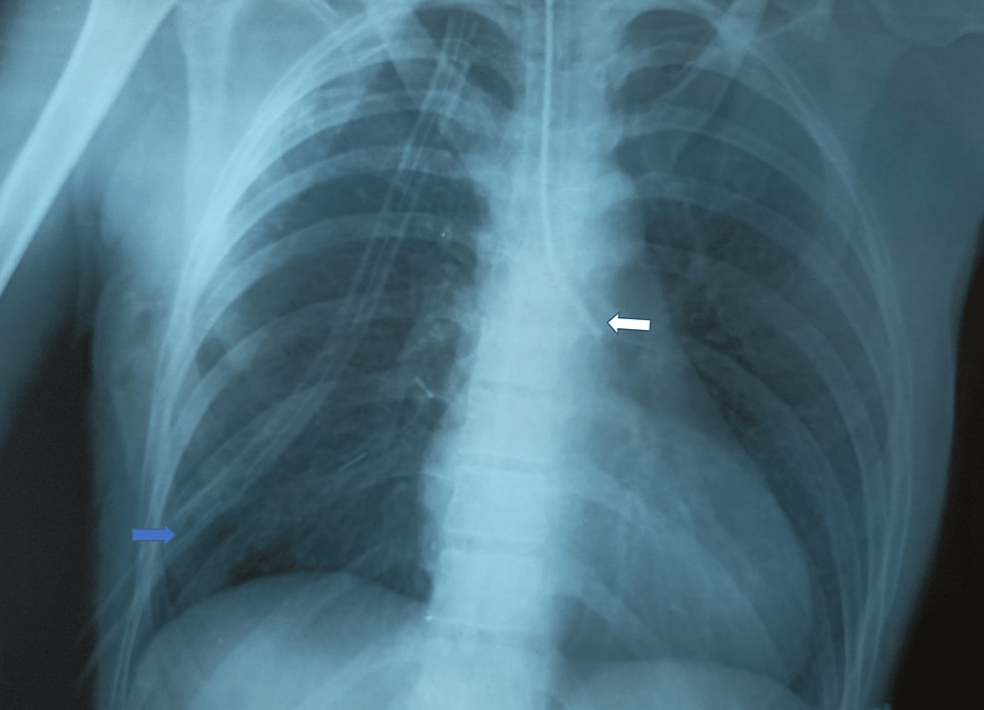
Chest radiograph showing the successful re-expansion of the right lung after chest tube's insertion (blue arrow). The white arrow represents an endotracheal tube placed in the operating room in order to isolate the left lung thus facilitating the expansion of the pneumothorax.

After an immediate thoracic-surgery consultation, a chest tube, with air water seal, was inserted in the theatre. The patient’s vital signs were immediately improved and remained stable (oxygen was completely recovered at a status of 98% saturation, blood pressure at 110/70mmHg, heart rate at 80 bpm, and RR at 12 breaths/min). The repeat chest radiograph revealed successful re-expansion of the right lung (Figure 2).

Three days later, the chest tube was removed and five days after the chest tube's removal, the patient was discharged from the hospital being completely recovered. She was examined one week later, after discharge, in the outpatient clinic department and she did not report any symptoms such as chest pain, dyspnea, and/or shortness of breath. The patient had a follow-up of four months, which was uneventful.

## Discussion

To the best of our knowledge, pulmonary complications arising from shoulder arthroscopic invasive procedures are not frequently described in the relevant literature [3,5]. Our patient had an unexpected pneumothorax after a routine procedure of rotator cuff repair, with no obvious or easily identifiable risk factors for the development of this respiratory complication. According to the literature, in most cases, it is difficult to determine the cause of such a complication with certainty [6]. The most prevalent factors include those related to anesthesia (general or regional) [7], technical issues (barotrauma, needle injuries, or pleural puncture), surgical procedures (patient positioning, continuous axial traction in LDP, equipment issues with pumps and suction devices/air entrainment, thermal forces induced by the diathermia) [8] as well as factors related to the patient (smoking habits, underlying lung disease, such as asthma, chronic obstructive pulmonary disease, emphysema, lung bullae, recent thorax trauma, prior history of pneumothorax, obesity, older age, and rare diseases such as alpha-1 antitrypsin deficiency) [9]. In our case, the patient was not obese, with no history of previous pulmonary disease, chest trauma, or smoking habits.

Regarding the correlation with general anesthesia without interscalene nerve blockade, only a few cases of pneumothorax, potentially associated with this type of anesthesia, have been described [10-12]. Numerous possible causes have been hypothesized, such as airway forces, bronchus intubation, and patient risk factors (smoking or lung diseases). The rupture of bullae or bleb seems to be the most common cause of pneumothorax during intubation [13,14]. Moreover, high trans-pulmonary pressure, implying barotrauma, can be attributed to difficult intubation associated with the use of airway exchange catheters or any assisted oxygenation tool with the administered gas being under pressure [11]. In our case, the patient remained stable throughout the procedure, and she was extubated uneventfully. The intubation was performed under direct immediate vision and no bullae were visible on the preoperative chest X-ray.

On the other hand, according to some studies, pulmonary complications occur during shoulder arthroscopy being attributed to the invasive procedure itself [15,16]. The commonest iatrogenic causes are considered to be the high pump pressure [17], changes in pressure in the subacromial space relative to atmospheric pressure, the large volume of irrigation fluids [18], the length of the operative procedure, interscalene nerve blockade technique [19], and the limited surgeon experience [20].

The exact pathogenetic mechanism for the entry of the air, in shoulder arthroscopy, is not clearly known. Nevertheless, it can be considered, that the transitional changes of the pressure emerged in the subacromial space throughout the whole invasive procedure, compared to the atmospheric pressure, facilitate the entry of the air through the lateral portals, causing possible injury to the pleura in association with the invasive handlings, thus leading to pneumothorax occurrence [21-23]. During the arthroscopic technique, the subacromial space is dilated, due to pressure exerted from the injected fluid with the use of an infusion pump, in order to establish a good field of view and most important an appropriate working space. The infusion pump works steadily to maintain a constant pressure. Frequent suction, applied from the instruments used to perform acromioplasty, results in transient falls of the pressure into the subacromial space which becomes negative compared to the atmospheric one. Negation of the pressure into the subacromial space results in air, inserted via lateral portals, trapping in the above space. Also, if the power shaver is turned off, then subacromial pressure becomes positive compared to the atmospheric one, pushing the air from the subacromial space into the surrounding tissue, thus resulting in subcutaneous emphysema [21,24].

Another debatable technical issue related to pulmonary distress is the patients’ positioning (lateral decubitus or beach chair) during arthroscopy. Orthopedic surgeons prefer either the LDP or the beach-chair position (BCP) to perform arthroscopic shoulder procedures with the aim of better visualization and easier access to the surgical field [25]. The LDP has been associated with the potential for peripheral neurapraxia, brachial plexopathy, direct nerve injury, and airway compromise, while the BCP has a higher rate of hypotensive, bradycardia, or cerebrovascular events and cervical neurapraxia [25].

Although the preferred position in most of the studies is the LDP, the authors hypothesized that the use of an arthroscopic pressure pump, combined with power-run shaving devices, was the main factor causing the complication. In our patient’s case, the operating position was also an LDP. However, to date, there is no objective evidence to support that any patient positioning is causally associated with respiratory complications during arthroscopic shoulder procedures [25].

Complications like pneumothorax, appearing in the sequel of invasive diagnostic procedures in the shoulder, such as arthroscopy, might be avoided on the condition that the following will be abided by the orthopedic surgeons: (i) proper placement of the portals, especially the placement of the anteroinferior portal, is considered of great importance; (ii) the outflow and inflow variations should be maintained in balance during the procedure, and the pump lavage fusion must be used as little as possible to avoid major subacromial pressure fluctuations; (iii) the use of sealing dams and arthroscopic cannulas during the procedure seems to prevent air entering through the portals. The preoperative suspicion of potential lung bullae and the prevention of airway trauma during intubation should be the main consideration of anaesthesiologists [25].

Also, the relevant literature reports potential and threatening-life complications arising from shoulder arthroscopy which must be taken into account by anaesthesiologists and surgeons [26]. Moreover, anatomically, the right lung is more developed compared to the left one. Thus, the message of our case is that lesions located in the right hemithorax, when diagnostic techniques are necessitated, can further contribute to the occurrence of complications like pneumothorax, therefore, the whole medical team should be alert when managing such a patient. Table 1 summarizes the cases of respiratory complications following shoulder arthroscopy.

**Table 1:** A literature review of pneumothorax complications after shoulder arthroscopy procedure. SLAP: superior labrum anterior posterior

Author	Age	Mechanism/Diagnosis	Type of Anesthesia	Positioning	Surgical Procedure	Complication(s)	Management	Possible Cause(s)
Bamps et al. [5]	42	Non mentioned	General	Lateral decubital	Diagnostic shoulder arthroscopy	Pneumothorax	Chest tube	Rupture of parietal pleura, related to the surgical methodology (portal placement and continuous pump infusion with intermittent suction) and subacromial distention used during the procedure
									Oldman [6]	41	SLAP lesion	General	Non mentioned	Arthroscopic subacromial Decompression/SLAP lesion repair	Small pneumothorax	Conservative management without drainage	None
Leander-Olsson et al. [7]	72	Fracture in fossa glenoidale and partial ruptures in subscapularis and supraspinatus.	Combined interscalene block with ultrasound guidance and general	Beach chair	Arthroscopic rotator cuff and fossa glenoidale fracture repair	Large pneumothorax and extensive subcutaneous emphysema	Chest-drain	Causes related to upper extremity block, general anesthesia, surgical procedure and patient factors									
									Shariyate et al. [11]	61	Massive rotator cuff tear	General	Beach chair	Shoulder arthroscopy/Rotator cuff repair	Pneumothorax associated with subcutaneous emphysema which expands to both the neck and the face	Chest tube	Three possible causes: the use of intra-articular shaving/related to general anesthesia/patient factors
Lee et al. [13]	45	Impingement syndrome	General	Beach chair	Arthroscopic subacromial decompression	Bilateral pneumothorax, extensive subcutaneous emphysema and pneumomediastinum	Chest tubes bilaterally	Transitional changes regarding the subacromial pressure’s values compared to the atmospheric ones, result in air’s insertion in the subacromial space via lateral portal. When the power shaver, connected to a suction device, stops working, the pressure in pump infusion becomes positive dispersing the air in the surrounding tissues, thus creating subcutaneous emphysema.									
									43	Impingement syndrome	General	Beach chair	Arthroscopic subacromial decompression	Extensive subcutaneous emphysema and pneumomediastinum	Gradually resolved
	40	Impingement syndrome	General	Beach chair	Arthroscopic subacromial decompression	Tension pneumothorax with extensive subcutaneous emphysema and pneumomediastinum.	Chest tube								
									Dietzel and Ciullo [14]	38	SLAP lesion	General	Lateral decubital	Shoulder arthroscopy/SLAP lesion debridement, and repair	Pneumothorax	Chest tube	Rupture of a bleb or bullae, associated with positive-pressure ventilation and underlying history of heavy smoking, asthma, or underlying lung disease.
	22	Electrocution dislocation/SLAP lesion	General	Lateral decubital	Shoulder arthroscopy/SLAP lesion debridement, and repair	Pneumothorax	Chest tube										
	37	Motor vehicle accident/labral tear	General	Lateral decubital	Labral tear’s debridement, subacromial decompression with acromioplasty	Pneumothorax	Chest tube										
34								Labral tear		General	Lateral decubital	Labral tear repair, subacromial decompression, imbrication of rotator cuff	Pneumothorax	Chest tube			
	Li et al. [15]	56	Full-thickness supraspinatus tear	Combined interscalene regional and general	Beach chair	Arthroscopic rotator cuff repair	Tension pneumothorax								Chest tube	Accidental damage of the prevertebral fascia, direct eating of the lung parenchyma, during block, leading to bullae ruptures attributed to positive pressure gradients created from ventilation supply when shoulder arthroscopy is performed	
																		Kim et al. [17]	75	Subscapularis tear	General	Non mentioned	Arthroscopic subscapularis repair	Pneumothorax and subcutaneous emphysema on the chest wall and neck	Chest tube	Extravasation of irrigating fluid
Tanoubi et al. [18]								63		Rotator cuff injury	Combined ultrasound-guided interscalene nerve block and general	Lateral decubital	Arthroscopic rotator cuff repair	Pneumothorax with subcutaneous emphysema and pneumomediastinum													Gradually resolved	Extravasation of irrigating fluid and air from arthroscopic system
	Bowden et al. [19]	30	Distal clavicle fracture	Combined ultrasound-guided interscalene nerve block and general	Beach chair	Arthroscopic acromioclavicular joint reconstruction and open distal clavicle resection	Large tension pneumothorax		Pigtail catheter						Ultrasound-guided interscalene nerve block anatomical investigation to perform shoulder arthroscopy, causes random perforation of the pleura and/or spontaneous pneumothorax													
Lau [21]								62		Glenoid labral tear	General	Non mentioned	Arthroscopic debridement of the glenoid labral tear	Pneumomediastinum and subcutaneous emphysema		Antibiotic therapy intravenously	Both saline solution and air were extravasated, resulting in subcutaneous emphysema. the subcutaneous air ruptures the prevertebral fascia, allowing air to enter the visceral space of the neck and subsequently the mediastinum
	Kim et al. [22]	86	Rotator cuff injury	General	Beach chair	Arthroscopic rotator cuff repair	Pneumomediastinum associated with subcutaneous and intermuscular emphysema with extension to both the face and neck		Administration of antitussive agent, antibiotics and 5 L/min of oxygen						Mild laceration in the oral cavity/soft tissue structure of the neck loosening by aging process		
	Calvisi et al. [23]	52	Subacromial impingement	Scalenic brachial plexus block	Beach chair	Arthroscopic acromioplasty and acromioclavicular osteophytectomy	Pneumomediastinum and subcutaneous emphysema		Spontaneously resolution						Puncture of the prevertebral fascia during scalene block, or high suction shaver, pump and outflow cannula can create a Bernoulli effect		
																		Our case	52	Rotator cuff injury	General	Lateral decubital	Arthroscopic rotator cuff repair	Pneumothorax	Chest tube	Maybe the positive pressure by the infusion pump during arthroscopy procedure

## Conclusions

Shoulder arthroscopy seems to be a safe and effective technique, based on a review of the literature. Complications like pneumothorax are very rare; however, they could be life-threatening for the patient. The surgical procedure by itself seems to be the most common cause. Portal placement, surgical technique, pump pressure, and continuous irrigation, rarely appear to cause this kind of complication. Traumatic intubation or preexisting pneumo-bullae are also possible causes of pneumothorax emergence. In any case, patients must be informed about the possibility of the later complication. Surgeons and anesthesiologists should be both prepared to deal with this rare event.
